# Hospital and Institutional News

**Published:** 1915-05-08

**Authors:** 


### HOSPITAL AND INSTITUTIONAL NEWS.

### MISHAP TO A HOSPITAL SHIP.

The hospital ship *Rewa* ran ashore on April 29 near May Island, but was eventually refloated and brought into Leith Harbour on Friday last week. It is very satisfactory to note that there were no wounded on board the vessel at the time of the accident, which is believed to have been caused by fog. No casualties of any kind occurred.

### ARMY CHAPLAIN AS X-RAY OPERATOR.

The Rev. C. E. Doudney, Vicar of St. Luke's Church, Bath, who recently went to the Front as a chaplain, is now stationed at one of the military hospitals in Rouen. He is well known in the West of England as an expert amateur electrician, and Mr. Doudney is putting his technical knowledge to practical use in the service of his country by combining *x*-ray work with his clerical duties.

### SHORTAGE OF RESIDENTS AT BRADFORD.

Owing to the absence of their two honorary assistant surgeons on Army service, and the difficulty of obtaining a sufficient number of resident medical officers, the Board of the Bradford Royal Infirmary have arranged with general practitioners in Bradford to assist in the wards, theatres, casualty and out-patient departments from 2 p.m. to 6 p.m. on Tuesday, Wednesday, Thursday, and Friday of each week. Each of the temporary assistants acts in conjunction with one of the four honorary surgeons, and it is hoped by this arrangement to maintain the work in full efficiency.

### THE GERMAN GAS.

The asphyxiating gas employed by the Germans in their recent advance on Ypres has been made the subject of a special investigation by Dr. J. S. Haldane, the brother of Lord Haldane. It is understood that the gas not only has an asphyxiating effect, but, judging from the after-history of the victims, also induces a form of blood-poisoning. The War Office's appeal to the general public for protective appliances, the suggested types of which we described in our last issue, was so successful that in twenty-four hours an announcement was made that a sufficient quantity had been obtained.

### A CONDITIONAL BEQUEST.

King Edward's Hospital Fund for London has an important prospective interest in the estate of the late Mr. Edward Berman, the gross value of which amounts to £95,888. The whole of the property is to be held in trust for his wife for life, and then for his son, and in the event of the failure of these trusts the principal part of the property, which amounts to some £60,000, is to be left to King Edward's Hospital Fund for London. This will is only another example of the great work which has been accomplished by the voluntary system in making the hospitals, as it were, the residuary legatee of all testators—the object, in a word, which they instinctively think of as a beneficiary in the event of other dispositions falling through. It has been in providing a special fund for this purpose that King Edward's foresight has been most characteristically shown.

### WAR PRESSURE AT CROYDON GENERAL HOSPITAL.

During the last few days twenty-three wounded soldiers—several of them from Hill 60—have been received at this hospital, and it is gratifying to learn that they all appear to be in excellent spirits. The hospital executive has offered accommodation for a further twenty-seven patients pending the completion of the military hospital in the Croydon district, for which several of the secondary and elementary Council schools have been requisitioned, with accommodation for 1,000 cases. In addition to this fifty beds set apart for military cases in the Croydon General Hospital, medical and surgical cases as they arise are received from the Naval Training School at the Crystal Palace. The reception and treatment of warrior patients means a great strain on the resources of the hospital, but it is felt that no effort must be spared to meet the national needs to the utmost.

### NEW DESIGNS IN RESPIRATORS.

The War Office demand for respirators, as we note elsewhere, has been met for the present, but separate mention should be made of two designs, approved by the authorities, which have been devised by Colonel Cantlie and Mr. Johnson of Allen and Hanburys. Colonel Cantlie's pattern, of which 2,000 have been ordered for the Grenadier Guards, is understood to be specially valuable in counteracting the fumes of bromine. It is made of a loose, mask-like fabric, fitted with a transparent slip of mica, and with an alkali filter at the mouth. The filter is added on the theory that an alkali is essential if the respirator is to be used more than once against bromine or chlorine. It is interesting to record that the surplus of the 300,000 respirators asked for by the War Office has been largely handed over to the Belgian Soldiers' Fund. Both the College of Ambulance and the Institute of Hygiene have sent their superfluity, which will be accepted until the Belgian Government issue a specification, which, at the time of writing, has not been the case.

### SCHOOLS USED AS HOSPITALS.

While we have from the first set our faces against the indiscriminate use of schools and municipal or local government buildings for hospital purposes, preferring the erection of temporary buildings on open sites, or the co-ordination of existing hospital structures, the adaptation of schools has proceeded to a certain extent. A recent official statement gave the number of elementary schools now in the hands of the military authorities as about 250 for England and Wales, which makes the number of school children who have been turned out 132,000. All except 6,000 children are stated to have been provided for by using neighbouring schools, which are attended by one set of children in the morning and by another in the afternoon. The problem of dealing with the evicted children is also giving a stimulus to the open-air school movement, the scholars being sometimes taught out of doors. Visits to museums are also being paid. In London five schools have been adapted to hospital purposes.

### WHY IS THE GUINEA SUBSCRIBER IN LONDON ASLEEP ?

It is claimed by the sceptical that figures can be made to prove anything. We venture to affirm that, thanks to the Uniform System of Hospital Accounts, and to its adoption by every well-managed voluntary hospital, hospital figures are probably the most accurate and reliable extant. So far as the London hospitals are concerned, this ought to be the precise truth, owing to the elaborate care and constant oversight and analysis now carried out by the King's Hospital Fund. Unfortunately, people outside the hospitals, or the majority of them, appear not to know or to have forgotten the existence of the King's Hospital Fund. The fact is that the King's Fund's income at present from annual subscriptions and donations is so small as to make their totals the most remarkable figures to be found in any report relating to hospitals the world over. The guinea subscriber, despite the example of their Majesties the King and Queen and that of their children, has yet to realise the existence and work of King Edward's Hospital Fund, 7 Walbrook, E.C., and to send his or her guinea to it every year.

### THE ANNUAL COST OF THE LONDON HOSPITALS.

Public speakers sometimes have very hazy views of the annual cost of maintaining the voluntary hospitals of London. Sir Andrew Wingate, speaking in North Paddington, as reported in the *Indicator,* stated that "the League of Mercy in fifteen years had collected £216,000 for the London hospitals. That meant at least that the League had for two years entirely supported the hospitals of London." The League of Mercy has given £216,000 to the London hospitals as stated, but that sum represents only one-thirteenth, or 7.75 per cent., of the total cost of their maintenance for two years. In 1913 the general and special voluntary hospitals in London contained 10,219 beds and expended, in round figures, just under £1,392,500. That cost is likely to be materially increased in 1915 by the admission of an ever-increasing number of sick and wounded warriors from the Front. The London voluntary hospitals are the backbone of the hospital and nursing provision for British sailors and soldiers wounded in the Great War. This fact should cause every thinking man and woman to send a contribution at least every year the war lasts to some London hospital, whatever other funds and fields of work they may support.

### A COMPETITION IN MEDICAL SALARIES.

The Southwark Board of Guardians have been landed in a difficulty by their failure to arrange a scale of adequate salaries for the medical staff of their infirmary. The Guardians of Camberwell were desirous of obtaining the services of an assistant medical officer, and acting on the advice of Dr. Keats, their medical superintendent, they decided to offer the greatly enhanced salary of £500 a year. Dr. Charles Witt, assistant medical officer at the Southwark Infirmary, applied for the post, and was appointed. His salary at Southwark was £200 a year, and on the very eve of his acceptance of the position at Camberwell the Southwark Guardians were proposing to increase it to £250 a year. Now that they have lost an assistant medical officer the Southwark Guardians have decided to invite applicants for the vacancy at a commencing salary of £250 a year, rising by annual increments of £25 to a maximum of £350. It is not business to treat the crisis in so tentative a manner, and we venture to doubt if such temporising tactics will bring success.

### A SUGGESTIVE RADIUM WEEK.

The institution of a Radium Week in connection with Northampton Hospital has been previously noted in our columns, and the latest report of its work shows that the local movement is progressing satisfactorily. With the end in view of properly equipping the hospital with radium, a committee was established to carry out the idea of a Radium Week. The second week in May was chosen, and Rose Day was held on the last day; while at the Town Hall was exhibited a model of Port Sunlight, the penny charge for viewing which brought £40 to the radium fund. Ten days after the week was over the Mayor and Mayoress invited the rose-sellers and workers in the towns and villages adjacent to a reception, when the official report of Radium Week was read. At this meeting the announcement was made that the total receipts and bank interest amounted to £3,116. After the payment of expenses, and the purchase of 150 milligrams of radium at a cost of £2,140 15s., there remains a balance of over £700, which will be used to procure more radium when the medical board of the hospital consider it to be required. These details are instructive as showing how such a week can be successfully organised, and may prove suggestive to other institutions contemplating the starting of a similar fund.

### THE MEDICAL OFFICER AND THE WORKHOUSE MASTER.

The Poor-Law Medical Officers' Supplement of *The Medical Officer* contains this month an account of the difficulties experienced by the former medical officer of a provincial workhouse in administering the sick wards, owing to the action of the master. The report states that "in one instance a dose of brandy, ordered for a patient seriously ill with pneumonia and who died the following morning, was refused by the storekeeper because the requisition for it was not presented at the usual time for issuing stores. In another instance a special diet, ordered for a patient suffering from gastritis, was discontinued by the master on his own authority, and was only renewed after an appeal had been made to the committee. Requisitions for such necessaries as bed-pan mops and for urgent cleaning or repairs required in the ward were either ignored or the execution of them delayed unwarrantably. Inmates who asked to see the medical officer were interviewed by the master, and in some cases, such as ulcers of the leg and bruises, examined by him, and, if he thought treatment unnecessary, were not brought before the doctor. Another constant source of grievance was that the master kept the imbecile wards short of inmate labour, although there were men and women in the house suitable for the work."

### L. G. B. ACTION CALLED FOR.

Unfortunately the cruel abuse of authority by masters of workhouses and other evil results following upon the system of workhouse administration adopted by the Local Government Board in the Poor-Law Institutions Order are far too frequent. From time to time fully authenticated reports of a master's delinquencies have reached us from workhouse medical officers in various parts of the country. The evil is undoubtedly widespread, and a revised system of workhouse administration is urgently needed. Any drastic alterations are for the time being owing to the war impossible. When the war is ended and the nation can again devote its attention to social reforms, the classification of workhouses so as to permit all the sick to be kept in separate institutions under medical control is a matter which will have to be pressed upon our legislators. Meantime, much can be done by medical officers to prevent such abuses as those complained of in the present instance. Where requisitions are refused or their execution unwarrantably delayed complaint should invariably be made to the visiting committee, and if redress is not obtained the complaint should be carried to the Local Government Board. Any interference with the treatment ordered should be similarly dealt with. Lastly, it is the duty of the medical officer to report to the Coroner a case such as that described, where a dying patient was refused brandy, so that a public investigation may be held into every similar instance of a master's brutal ineptitude.

### THE REGISTRAR-GENERAL AND MORTALITY STATISTICS.

Several facts of interest in reference to disease emerge from the Report of the Registrar-General on Births, Deaths, and Marriages in England and Wales in 1913, which was issued as a Blue-book last week. The delay in its appearance is set down to the depletion of the experienced office staff, combined with the new duties which are being imposed on those who remain behind. The analysis of the statistics notes that the death-rates of phthisis and tuberculosis as a whole were the lowest on record, and generally that the mortality from diseases of the lungs shared this satisfactory decline. Indeed, mortality from the chief epidemic diseases is recorded as below the average, while the mortality in particular from enteric fever and whooping cough was the lowest on record. Against this must be set the fact that cancer is recorded as causing a higher death-rate both among males and females than in any preceding year. A good example of the caution which must be exercised in deducing generalisations from statistics is shown in connection with the table which indicates the incidence of cancer mortality in London, county boroughs, urban areas, and rural districts. This table apparently indicates a constant increase in standardised mortality in proportion to urbanisation, but the obvious reminder is added that this result may be due to the better facilities for diagnosis which are available in urban areas. Cancer of the breast is stated to be increasing, and uterine cancer to be diminishing.

### THE LONDON HOSPITAL AT THE ROYAL ACADEMY.

A visit to the Royal Academy in peace-time rarely reveals hospital subjects, although the year's portraits include, as a rule, those of prominent physicians and surgeons, and perhaps a storied canvas on some consulting-room theme. This year, however, is an exception, and institutional workers will not fail to note with gratification that the work for the wounded which is being performed on such a wonderful scale by the voluntary hospitals of the country has provided a motive for art which will become historic in the sense of epitomising a great national activity. We refer, of course, to Mr. Lavery's picture, "Wounded: London Hospital, 1915," marked 181, on which, as already recorded in The Hospital, the artist has been at work during the earlier months of the year. Historic subjects, however, are apt to overweigh the artist, and Mr. Lavery has perhaps not wholly subdued the historic element to his imagination. Thus the nurse and wounded soldier in the foreground strike the motive, and the painter seems to be more at ease with the interior of the ward, which is the real artistic subject-matter of the picture. It is necessary to emphasise this point, since a picture will live for its imagination, composition, colour, and drawing, defects in which can never compensate for the historic interest of its subject-matter. On the other hand, an artist is as free to find his motive in a hospital ward as anywhere, and the London Hospital may be congratulated on having in Mr. Lavery a distinguished artist to record the great work which, in common with other hospitals, it is performing at the present critical time.

### A DOCTOR'S FEAR OF PREMATURE CREMATION.

While we all know of cases in which a fear of premature burial is strong, it is much more rare to meet with a fear of premature cremation. Indeed, one of the arguments most frequently used in support of cremation is the small risk of mistakes as to the fact of death which is secured by the elaborate certification imposed by law on those who wish for this form of burial. Yet the will of a medical practitioner, Dr. Arthur Wigelsworth Orwin, of Minehead, shows that the fear of premature cremation can be very strong. The following direction may be quoted in full:—

That until it was ascertained that he was in fact dead his nose and mouth were not to be covered nor his body confined in any shell or coffin; that on his apparent death his body should be kept in a well-warmed bed for thirty-six hours and then placed in a warm room, with the windows partially opened, and watched for twelve days and nights or until definite signs of decomposition have set in. During this period the tests given in a pamphlet by Sir Benjamin Ward Richardson, "The Signs and Proof of Death," were to be applied, and a bell was to be attached to his wrist. When decomposition should have set in, a surgeon was to sever completely the spinal cord high up in the body, which was then to be cremated.

Dr. Orwin, who was consulting physician to the Central London Ear and Throat Hospital, left estate of the gross value of £93,512. He bequeathed £1,000 to this institution, and £100 each to five other charities.

### RETURN FROM THE NAVAL SERVICE.

The Lambeth Board of Guardians have lost by resignation the services of Dr. G. S. Miller, which has deprived the staff of the only medical officer who specialised in surgery. To meet the difficulty arrangements have been made with the Admiralty by which Dr. G. F. Stebbing has been allowed to resign his position as temporary fleet surgeon, on condition that Dr. Bowen, the second assistant medical officer, shall replace him. Dr. Stebbing incurs considerable financial loss by this arrangement, but he prefers the work at the infirmary, and recognises, so Dr. Baly informed the board, that he is fulfilling a more useful purpose by returning to the infirmary. The work at the infirmary has grown considerably through the conversion of Lewisham Infirmary into a military hospital for wounded and sick soldiers, no fewer than 101 patients from Lewisham having been accommodated at Lambeth. The additional demands on the medical and nursing staffs thus caused will have to be met, despite the spirit of loyal co-operation amongst the Poor-Law authorities of the Metropolis which has removed many difficulties. Mr. Frank Briant, L.C.C., warned his colleagues at the Lambeth Board of Guardians that it was not unlikely they would have to provide accommodation for wounded and sick soldiers in their infirmary. In our view the Guardians would be well advised to prepare in advance to meet such an emergency with efficiency and promptitude.

### METROPOLITAN ASYLUMS BOARD AND THE PROVISION OF SANATORIA.

The conference called by the Wandsworth Board of Guardians to take action against the Metropolitan Asylums Board to compel them not to take from the poor-rate the money they require for the erection of sanatoria for the accommodation of phthisical cases ended in a decision being arrived at to appoint a deputation to wait upon the Local Government Board. It was generally felt that the money had to be found by some authority or other, but it would have been a much honester course if the London County Council had dealt directly with the matter rather than hand their powers over to an authority who appeal directly to the Poor-Law bodies for their monetary assistance. The resolution carried by the conference was "that delegates be appointed to wait on the Local Government Board to enter a protest against sanatoria benefits for other than Poor-Law patients being made a charge on the Poor-Law funds, and not paid out of Government or county funds, as is directed by Clause 64 of the National Insurance Act, 1911." Until the whole facts are laid before the Local Government Board it would be unwise for any authority to take legal action, and it is doubtful if it could be fought to a success in view of the Amending Act, which gave the London County Council the power to enter into an agreement with another authority to provide the necessary sanatoria for phthisical patients in London. It is also doubtful whether the Local Government Board would countenance the commencement of litigation against the Metropolitan Asylums Board, which might be fruitless as well as costly.

### INCOMPETENT LOCUM TENENS.

The Lambeth Infirmary has been seriously depleted of its medical staff, three assistant medical officers having joined the Forces. After making inquiries as to the results experienced in other institutions, Dr. Baly, the medical superintendent, has informed the board that he has come to the conclusion that it would be better to work with a reduced staff than to pay the excessive price demanded by a *locum tenens*, "mostly incompetent." He had obtained the services of two unqualified clinical assistants, who have relieved the three remaining assistant medical officers of their less responsible duties. He had received an application from Mr. B. A. Keats asking that he may be paid for the duration of the war at the same rate as the *locum tenens*—namely, six guineas a week. Mr. Keats could easily obtain employment either in the Army or elsewhere at a higher rate, and Dr. Baly thought it would be impossible to obtain a qualified medical man to perform the particular duties which he had to do. He further pointed out that Mr. Keats had performed many extra duties over and above his ordinary work. The infirmary visiting committee pointed out that the board was well aware of the extreme difficulties which had been experienced in obtaining the services of qualified medical men to undertake the institutional and other medical work of the parish. So although the salary now suggested to be paid to Mr. Keats represented a large increase, it was not disproportionate in view of the fact that he would have no difficulty in securing the same elsewhere should he be so disposed. Acting on the advice of the committee, the Guardians decided to pay Mr. Keats the salary suggested by Dr. Baly, having regard to the loyal service he had rendered through the onerous period during which the medical staff has been inadequate.

### THE RED CROSS AND THE COMITY OF NATIONS.

Attention has been drawn to the good work for the Allied cause, in other than a medical sense, which is being done by the Red Cross workers.

As the statement of the good will thus brought about was made by an Englishman returned from France, he naturally emphasised what his experiences had taught him in regard to the work of English doctors, nurses, and orderlies for their French and Belgian comrades. But it would be manifestly unjust and incorrect to draw the line here. Thus it becomes interesting in this connection to study the effect of the Japanese Red Cross Hospital in Paris, where the Rising Sun and the Red Cross appear side by side. This hospital, whose equipment, which is said to be up to date in every respect, has come from Japan, should do much to improve mutual understanding between the French and the Japanese from the fact that while its staff includes Japanese nurses, their ignorance of French and English has led to the actual nursing being undertaken by the French Red Cross workers. The Japanese, however, are actively employed in the preparation of dressings, and such work as is not actually involved in bedside ministrations. It is, therefore, clear that these representatives of the Eastern and Western civilisations will begin to come to some appreciation of each other's qualities, for which active co-operation in common work is the best possible means. Satisfaction at this result should come home especially to institutional workers, whose system of voluntary personal service is thus adding a new wreath to its laurels. It is helping to bind together different nations as surely as the war has divided them, and we may believe that the barriers which it is breaking down in the stress of a common work caused by the war will not be wholly able to reassert themselves when peace is declared, to the lasting gain of civilisation.

### THE FIRST POOR-LAW CONVALESCENT HOME.

Progress in Poor-Law work is being brought about in many useful directions, and while the most important step must be for a long time yet the amalgamation of small Unions, in such centres as Birmingham the public spirit of which this centralisation is the outcome manifests itself in other ways. A typical example which has accompanied the Birmingham amalgamation is the opening of a convalescent home at Wassell Grove House for the benefit of patients from the Birmingham Poor-Law infirmaries. This is the first institution in the kingdom to be opened under Poor-Law management, and the importance of the new departure is obvious. In 1913 a small committee was appointed to find premises, and in July of last year the new building, which was at the time the residence of the Mayor of Dudley, was secured for a rent of £135 per annum on a lease of twenty-one years. The accommodation provides for some forty patients, and the house is to be used for male patients from the three infirmaries. It is a great tribute to the public spirit of Birmingham, so often a pioneer in the past, to possess the first convalescent home attached to a Poor-Law Union, and we hope that their action will be copied in other parts of the country. But it is well also to remind enthusiasts that such new departures are practically impossible so long as the present wasteful decentralisation of Unions and institutions exists. Economy, classification, and convalescent homes for Poor-Law patients are the fruits of amalgamation; and it is, therefore, inevitable that the first Poor-Law convalescent home should have been established in the centre which was the first to enter upon a comprehensive amalgamation of the Unions. Manchester, so lately started on a similar amalgamation scheme, may be expected to show similar developments before long.

### POOR-LAW MEDICAL OFFICERS AND ATTENDANCE ON THE WOUNDED.

At the recent meeting of the Council of the Poor-Law Medical Officers' Association attention was drawn to the fact that in many instances the work of Poor-Law medical officers had been considerably increased by the number of sick and wounded soldiers who are being treated in Poor-Law institutions. In some cases unoccupied wards have been filled with invalided soldiers; in others the number of inmates has been considerably increased by the reception of patients from Boards of Guardians whose institutions have been taken over entirely by the War Office. In practically all cases this extra work had been thrust upon medical officers without any consultation with them, and without any extra remuneration even in those cases where the War Office was paying the Guardians a sum which included payment for medical attendance. The Council felt that those medical officers who were receiving no recognition for their services in the form of military rank had a distinct grievance. The extra work meant that Poor-Law medical officers would have to sacrifice practically the whole of their leisure time. It was recognised that they would do this without murmuring; nevertheless, it was felt that their patriotism was deserving of some return. The following resolutions were unanimously adopted, and it was decided to forward copies to the Local Government Board:—" (1) That in all cases where military duties are discharged by Poor-Law medical officers the salaries paid to whole-time officers should be on a scale not less than that paid by the War Office to members of the Royal Army Medical Corps discharging similar duties. (2) That in the case of part-time medical officers an addition should be made to their salaries commensurate with the extra labour involved. (3) That a medical officer placed in charge of a military hospital should receive the military rank usually given to a member of the Royal Army Medical Corps in charge of a hospital of the same size."

### A COMPETITION IN HUMAN SKINS.

Any unusual use for an object excites public interest, and any unusual use for the human skin has always attracted certain classes of people. For instance, we have all heard of the horrid attraction which human skin has for certain bibliophiles, and a book professedly thus bound has probably been shown to some of our readers. When people have an opportunity of sacrificing a portion of their own skin for a scientific purpose the attraction is increased, and that the modern operation of skin-grafting can readily find volunteers is shown by the answers mentioned in the *Times* to the following unusual advertisement which appeared lately in its columns: "Skin.—Officer requires 4 in. by 3 in. of skin to cover wound and expedite his return to duty; opportunity for unselfish patriot." The journal, in recording the fact that more than sixty offers were received from "unselfish patriots," discloses the fact that the advertiser is an Army airman, "at present in a London hospital recovering from injuries received in a flying accident several weeks ago." It is stated also that the majority of the replies were received from women anxious "to do their bit," as the phrase goes, and the respondents include "a girl of twenty-five," "a boy of seventeen," and "a man of forty-five"; the last-mentioned offers also his "blood." The replies show varying degrees of responsibility and good sense, and we are glad to note that one is quoted in which are required "a guarantee of your need and particulars of the hospital and surgeon who would perform the operation." This letter is written apparently by the headmistress of a girls' school, and if the writer is passed by a responsible doctor as fit her offer may claim serious consideration. We rather question, however, the desirability of such advertisements, since for an operation of the kind a suitable candidate should be found privately, and a hospital should be able to find a fit volunteer in the ranks of those within the circle of its influence.

### THE GERMAN HOSPITAL SHIP CASE.

The hearing of a remarkable case concerning the German hospital ship *Ophelia* began last Tuesday before the President of the Probate, Divorce, and Admiralty Division. The hospital ship, it will be recalled, was brought into Yarmouth by H.M.S. *Meteor* on October 18 last. The claim of the Crown was that the vessel's movements were so suspicious that she was not entitled to immunity from capture, which a hospital ship can claim under the Hague Convention. The Attorney-General, the Solicitor-General, and Mr. C. R. Dunlop appeared for the Crown, and Staff Surgeon J. V. C. Pfeiffer, of the German Navy, who is claimant on behalf of the Imperial German Government, was also represented by counsel. The allegation was that the *Ophelia*, though ostensibly a hospital ship, was actually engaged in scouting duties. The Attorney-General, in opening the case, said that when the vessel was seized on October 18 the log which was then taken confirmed previous suspicions. On August 3 she was in the Port of London, when she received orders to steam to a German port "for military duties." She sailed on the 4th, and after being refitted as a hospital ship on August 14 was taken charge of by Pfeiffer. The certificate of the use of the vessel, dated September 14, was then read, but the question was what actual use was made of her. A chart of her movements, according to her log, from September 18 to October 18 was then shown. The affidavit of Lieutenant-Commander Moncrieffe, R.N., of British Submarine D4, which had the vessel under observation on October 8, was read, in which the hoisting and hauling down of her Red Cross flag was described, and suggested to Lieutenant-Commander Moncrieffe that it was a signal employed by her on sighting his submarine. His opinion was that she was engaged in scouting. The affidavit of Lieutenant F. T. Peters, R.N., of H.M.S. *Meteor*, who sighted the *Ophelia* on October 18, was read describing his search of the vessel, his suspicions based on the vagueness of the orders given to the vessel, and his instructions to dismantle the wireless telegraphy apparatus, and to order her to follow him into port. Another affidavit was that of Commander Edward J. H. Newman. He inspected the vessel last December, under the impression that the British Government proposed to purchase it as a hospital ship. He reported that she was quite unsuitable owing to the lack of scuttles, lack of sanitation, and lack of accommodation for the staff. The only way to get the sick to the wards was by hoisting by derrick after taking off the hatchway. The Attorney-General concluded by submitting, after further evidence, that the *Ophelia* was clearly used as a signalling ship. The claimant's contention is that the case is one of mere suspicion, and it is still proceeding.

### THIS WEEK'S DRUG MARKET.

The most important question of the week is that of the proposed duty on spirits, and in view of the uncertainty as to whether or not the increased duty on spirits, if confirmed by the House of Commons, will apply to spirits used in the manufacture of pharmaceutical preparations the position is a difficult one, and if the full tax is imposed on medicinal spirits there will, of course, be a vast increase in the price of a large number of preparations. The drug market, generally, continues active. Prices of the salicylates and of most of the synthetic drugs are firmly maintained. English makers of bromides have advanced their prices, and it is probable that the limit has not been reached. The shortage of Epsom salts is becoming more noticeable. Gum benzoin is dearer. Belladonna leaves and roots have again advanced in price, and their preparations are dearer in consequence. Olive oil has an upward tendency in price, while santonin has a lower tendency. Quinine prices have an upward tendency. Balsam of Peru, which is in great demand, is still advancing in price. Russian cantharides are somewhat cheaper. There is little change in the position of cod-liver oil. Opium is steady. Thymol is dearer. English valerian root is very dear and scarce.

### TO OUR READERS.

Contributions are specially invited from any of our readers to these columns. They should deal with topical subjects and news. They must be authenticated for the information of the Editor only. The minimum payment if published is 5s. There is no hard-and-fast rule as to space, but notes of about twenty lines in length are preferred.

